# Diagnosing acute lower respiratory tract infections in out-of-hours services during the COVID-19 pandemic

**DOI:** 10.1186/s12245-025-00942-z

**Published:** 2025-07-30

**Authors:** Bent Håkan Lindberg, Beatriz González López-Valcárcel, Jonas K. Olsen, Malene Plejdrup Hansen, Jesper Lykkegaard, Carl Llor, Lina Jaruseviciene, Pascale Bruno Bazureault, Maria-Nefeli Karkana, Ana García-Sangenís, Anna Kowalczyk, Ingrid Rebnord

**Affiliations:** 1 National Centre for Emergency Primary Health Care, NORCE Norwegian Research Centre AS, Nygårdstangen, PO Box 22, Bergen, N-5838 Norway; 2 Dep. of General Practice, Inst. of Health and Society, University of Oslo, Oslo, Norway; 3 University of Las Palmas de Gran Canaria, Las Palmas, Spain; 4 Research Unit of General Practice, Department of Public Health, University of Southern Denmark, Aarhus, Denmark; 5 Center for General Practice, Aalborg University, Aalborg, Denmark; 6 Institut Català de la Salut, Barcelona, Spain; 7 Department of Family Medicine, Lithuanian University of Health Sciences, Kaunas, Lithuania; 8 Department of Public Health, Nice University Hospital, Nice, France; 9 Clinic of Social and Family Medicine, School of Medicine, University of Crete, Heraklion, Greece; 10 Fundació Institut Universitari per a la Recerca a l’Atenció Primària de Salut Jordi Gol, Barcelona, Spain; 11 Centre for Family and Community Medicine, Faculty of Health Sciences, the Medical University of Lodz, Lodz, Poland; 12 Department of Global Public Health and Primary Care, University of Bergen, Bergen, Norway

**Keywords:** Primary care, Out-of-hours, Respiratory tract infections, Pneumonia, Diagnostic process

## Abstract

**Background:**

Acute lower respiratory tract infections (LRTIs) commonly lead people to seek out-of-hours primary care. Symptoms of lower respiratory tract infections overlap, and access to definite diagnostic tools is lacking in most out-of-hours settings. Distinguishing between different LRTIs is vital to ensure appropriate antibiotic prescribing.

The study aimed to identify which clinical factors have guided out-of-hours physicians in distinguishing LRTIs in the late phase of the COVID-19 pandemic.

**Methods:**

Out-of-hours physicians from France, Greece, Lithuania, Poland, and Spain registered pre-defined clinical information about all cases suspected of an RTI on an A4-paper Audit Project Odense chart from January to March 2022. Two multivariable logistic regressions were performed to analyse which clinical factors the physicians used to distinguish between pneumonia and other LRTIs.

**Results:**

A total of 1,222 cases of either pneumonia, acute bronchitis/bronchiolitis, common cold/influenza, or COVID-19 were registered by 86 participating physicians. Fever and cough were the most common symptoms reported. The pneumonia diagnosis was associated with abnormal lung auscultation (odds ratio (OR) 11.41, 95% confidence interval (CI) 4.14–31.45), poor general condition (OR 5.96, CI 2.43–14.60), tachypnoea (OR 2.55, CI 1.38–4.73), and a combination of fever and cough (OR 11.10, CI 2.87–42.97).

**Conclusion:**

During the late COVID-19 pandemic, out-of-hours physicians’ registered information about the patients’ clinical condition, respiratory rate assessment, and lung auscultation evaluation were associated with diagnosing pneumonia, among other LRTIs.

**Supplementary Information:**

The online version contains supplementary material available at 10.1186/s12245-025-00942-z.

## Introduction

Symptoms of acute lower respiratory tract infections (LRTIs) are common reasons for encounters in out-of-hours services, including emergency primary care and emergency departments [1, 2]. Most LRTIs have low urgency, are self-limiting, and do not require antibiotic treatment regardless of viral or bacterial aetiology [3]. Nevertheless, a substantial percentage of patients presenting to out-of-hours services with LRTI symptoms receive an antibiotic prescription [4–6].

Overlapping symptoms and signs makes separating LRTIs like pneumonia, bronchitis, influenza, and, to a certain degree, even the common cold, a clinical challenge, especially in settings with limited diagnostic tools. Moreover, this distinction is essential to ensure appropriate antibiotic prescribing, as pneumonia is generally the only antibiotic-demanding infection.

The most common pneumonia symptoms and clinical signs include cough, dyspnoea, fever, fatigue, tachypnoea, and abnormal lung auscultation, all of which may be present even in other LRTIs [7]. The COVID-19 pandemic added a new diagnosis to the possible LRTIs with these symptoms.

Access to accurate diagnostic modalities is lacking in most primary care out-of-hours settings, and even the widely available SARS-CoV-2 point-of-care (POCT) to identify cases of COVID-19 have low sensitivity and specificity [8, 9]. C-reactive protein (CRP) POCT may help distinguish between pneumonia and self-limiting LRTIs with a similar clinical presentation [10]. However, no symptoms, signs or POCT results are definite for the diagnosis, and the overall clinical impression has higher diagnostic accuracy than clinical decision rules [11].

Out-of-hours services, characterised by unknown patients with higher urgency and lack of follow-up, carried a substantial burden regarding organisational changes and clinical handling of the COVID-19 pandemic [12, 13]. Nevertheless, how out-of-hours physicians diagnosed pneumonia among other LRTIs during this demanding period has not been investigated.

The present study was conducted in five European countries with a comparable prevalence of infectious diseases [14]. It aimed to identify which clinical factors have guided out-of-hours physicians in distinguishing pneumonia from acute bronchitis/bronchiolitis, common cold/influenza, and COVID-19 from January to March 2022.

## Methods

### Design and setting

Health Alliance for Prudent Prescribing and Yield of Antibiotics in a patient-centred perspective (HAPPY PATIENT) is a European Union-funded project with 15 partners within nine countries [15]. Out-of-hours physicians from France, Greece, Lithuania, Poland, and Spain registered on A4-paper APO charts the essential features of all consultations regarding community-acquired infections before and after a multifaceted intervention (see Appendix A) [16]. The present study used data from the pre-intervention audit.

The structure of out-of-hours services in European countries varies [17]. The five countries in this study also have different ways of organising out-of-hours care. Lithuania provides out-of-hours service in hospital emergency departments, mainly by hospital doctors. The other four countries employ general practitioners (GPs) in emergency departments and primary care centres of varying sizes and organisations. However, all the clinics included are the first point of contact for the population when a perceived medical need arises out of hours.

Data collection occurred before any possible referral for X-rays, ultrasound or additional blood tests available in nearby or linked hospitals, supporting comparability between countries and settings. Hence, despite variations in provision locations, organisational structures, and participants’ medical backgrounds, all data were uniformly analysed.

### Subjects and data

The APO chart was developed based on evidence, prior APO studies, pilot-testing, and consensus-seeking [7, 15]. It was pilot-tested in all five countries and reported as face-valid and sufficiently exhaustive after dialogue between the country representatives and the out-of-hours physician testers.

The participating physicians registered patients’ age and sex, ticked off their symptoms and clinical findings, POCTs used, which diagnosis the physician deemed most likely, and if antibiotic treatment was initiated. POCT results were not registered. Information about each participating physician was obtained digitally at sign-up for the study.

### Statistical analyses

Frequencies, percentages, means, and interquartile ranges (IQR) were used to describe the distribution of the continuous variables in the sample. A binomial logistic multilevel model, whose dependent variable is the pneumonia diagnosis (yes/no), was estimated. The patient was level 1, and the physician was level 2 (also called group).

Due to the diversity of symptoms related to COVID-19, all symptoms and clinical findings from the APO chart associated with RTIs (i.e., fever, sore throat, cough, increased sputum, ear pain, other symptoms, poor general condition, tachypnoea, abnormal lung auscultation, tonsillar exudates, and tender cervical adenopathy) were initially considered as potential predictors and an iterative forward selection method of regressors was performed [18]. For further analyses, variables with p-values less than 0.2 and those deemed clinically meaningful a priori (i.e., all except sore throat, ear pain, tonsillar exudates, and tender cervical adenopathy) were kept. Hence, age groups, fever, sore throat, cough, increased sputum, other symptoms, poor general condition, tachypnoea, and abnormal lung auscultation were independent variables in the regression. As the risk of severe pneumonia outcomes increases with age, the population was divided into age groups (0–17, 18–64, and 65+) [19]. A missing age category was included to keep all patients in the model estimation.

Based on clinical experience, we specifically wanted to test whether the following three combinations of symptoms and signs were associated with the pneumonia diagnosis: fever and cough, fever and abnormal lung auscultation, and poor general condition and abnormal lung auscultation. Hence, two regression models with the same variables were performed. Model 1 (M1) was run without the symptom combinations, while model 2 (M2) included the combinations. For both models, all the cases diagnosed with a combination of pneumonia and COVID-19 were excluded.

Stata 17 (StataCorp. 2021. Stata Statistical Software: Release 17. College Station, TX: StataCorp LLC) was used for the statistical analyses.

## Results

Eighty-six out-of-hours physicians from France, Greece, Lithuania, Poland, and Spain participated. The physicians had a median age of 35 years (range 25–64), and 61% were female. General practitioners (48%) and emergency medicine specialists (24%) comprised the largest group. The predominant model was consultations at a clinic (89%), and only 6% of the clinics had nurse-led telephone triage.

A total of 1,222 patients clinically diagnosed with either pneumonia (20.1%), COVID-19 (35.2%), common cold/influenza (37.4%), or acute bronchiolitis/bronchitis (15.3%) were registered (Table 1). Multiple diagnoses were provided for 98 cases (8.0%), of whom 78 were diagnosed with COVID-19 and pneumonia. Lithuanian physicians diagnosed pneumonia and COVID-19 more often than physicians from the other countries.

**Table 1:** Number of registered diagnoses per country (% of all included diagnoses)*

Country	Acute Bronchitis/bronchiolitis *N* (%)	Common cold/influenza *N* (%)	COVID-19 *N* (%)	Pneumonia *N* (%)	Total *N* (%)
France	6 (0.5)	21 (1.7)	19 (1.6)	14 (1.2)	60 (4.9)
Greece	24 (2.0)	20 (1.6)	38 (3.1)	20 (1.6)	102 (8.3)
Lithuania	77 (6.3)	125 (10.3)	225 (18.4)	167 (13.8)	594 (48.6)
Poland	44 (3.6)	146 (12.0)	71 (5.8)	27 (2.2)	288 (23.6)
Spain	36 (3.0)	145 (11.9)	77 (6.3)	18 (1.5)	276 (22.6)
Total	187 (15.3)	457 (37.4)	430 (35.2)	246 (20.1)	1320 (108.0)*

*Footnote: 98 patients across all age groups received more than one diagnosis

The patients had a median age of 40 (IQR 18–66), 27% were 65 years or older, and 48% were male (Table 2). The median symptom duration was four days for pneumonia (IQR 3–6), three days for COVID-19 (IQR 2–6), and three days for the other included LRTIs (IQR 2–4).

**Fig. 1:**
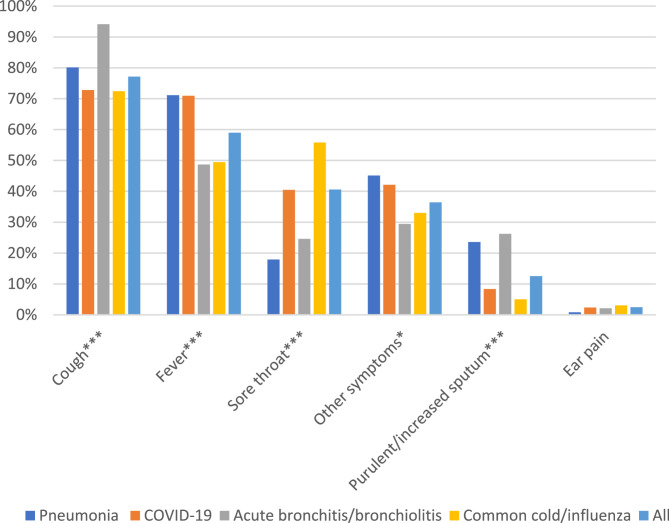
Lower respiratory tract infections: Symptoms in 1222 patients in European out-of-hours services. Legend: The percentage of cases registered with the listed symptoms. *=*P* ≤ 0.05, ***= *P* ≤ 0.001(Chi Square test)

**Fig. 2:**
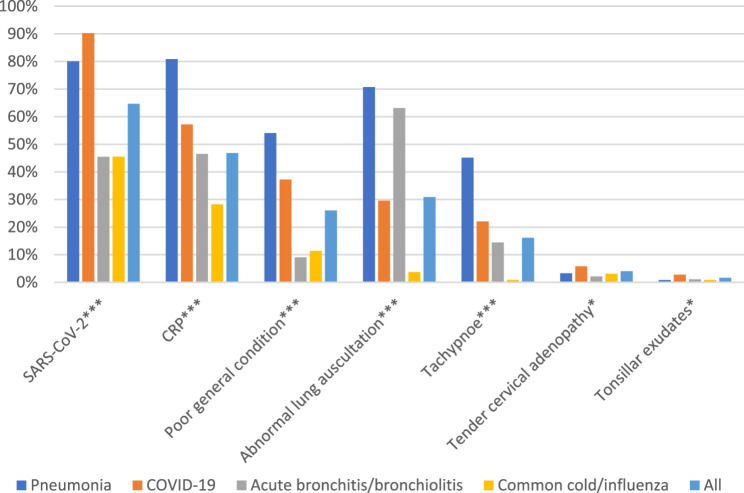
Respiratory tract infections: The use of POCTs and clinical findings in 1222 patients in European out-of-hours services. Legend: The percentage of cases registered with the use of POCTs and the listed clinical findings. *=*P* ≤ 0.05, ***= *P* ≤ 0.001(Chi Square test)

Cough (76.9%) and fever (59.0%) were the most common symptoms regardless of diagnosis (Fig. 1). Abnormal lung auscultation (30.9%) and poor general condition (26.0%) were the two most common clinical findings (Fig. 2). A combination of fever and cough was more frequently reported for patients diagnosed with pneumonia than with the other LRTIs (Table 2). Nine patients diagnosed with pneumonia were registered with cough as the only symptom.

**Table 2:** Multivariable two-level logistic regressions to identify factors associated with diagnosing pneumonia among relevant differential diagnoses in European out-of-hours services

	Acute bronchitis/bronchiolitis, common cold/influenza and COVID-19 *n* (%) (a,b)	Pneumonia *n* (%) (a,b)	Odds ratio (95% CI) in M1 (the regression model without combinations of signs/symptoms)	Odds ratio (95% CI) in M2 (the regression model with combinations of signs/symptoms)
Age group				
0–17	276 (28.3)	18 (10.7)	1 (ref)	1 (ref)
18–64	510 (52.3)	56 (33.3)	1.74 (0.68–4.42)	1.57 (0.61–4.06)
65+	176 (18.0)	93 (55.4)	3.86** (1.48–10.08)	3.74**(1.41–9.89)
Missing	14 (1.4)	1 (0.6)	1.58 (0.12–20.42)	1.75 (0.12–24.57)
Total	976 (100.0)	168 (100.0)		
Symptoms				
Fever	546 (55.9)	117 (69.6)	2.25**(1.28–3.94)	0.39 (0.11–1.39)
Sore throat/throat pain	452 (46.3)	29 (17.3)	0.29***(0.15–0.55)	0.27***(0.14–0.52)
Cough	742 (76.0)	137 (81.5)	1.40 (0.71–2.78)	0.32* (0.11–0.91)
Increased/purulent sputum	95 (9.7)	45 (26.8)	1.72 (0.91–3.27)	1.78 (0.93–3.43)
Other symptoms^c^	334 (34.2)	62 (36.9)	0.87 (0.49–1.55)	0.76 (0.42–1.37)
Clinical findings				
Tonsillar exudates	18 (1.8)	2 (0.0)	3.40 (0.50-23.05)	3.12 (0.42–23.14)
Poor general condition	185 (19.0)	91 (54.2)	3.76***(2.00-7.08)	5.96***(2.43–14.60)
Tachypnoea	86 (8.8)	71 (42.3)	2.52**(1.38–4.62)	2.55** (1.38–4.73)
Abnormal lung auscultation	203 (20.8)	120 (71.4)	7.19***(4.12–12.56)	11.41*** (4.14–31.45)
Combinations				
Fever and cough	440 (45.1)	103 (61.3)	**-**	11.10***(2.87–42.97)
Fever and abnormal lung auscultation	156 (15.6)	85 (50.6)	-	0.75 (0.25–2.22)
Poor general condition and abnormal lung auscultation	87 (8.9)	67 (39.9)	-	0.53 (0.18–1.55)
Group variable				
Physician (Median Odds Ratio)			**4.42**	**4.50**

*=P ≤ 0.05, **=P ≤ 0.01, ***= P ≤ 0.001

^a^78 cases diagnosed with both pneumonia and COVID-19 were excluded

_b_20 cases with other combinations of diagnoses were included

^c^“Other symptoms” was a choice on the APO chart

A CRP test was performed in 46.8% of the consultations, and a SARS-Cov-2 POCT in 64.7% (Fig. 2). Where the physicians diagnosed pneumonia, the SARS-CoV-2 POCT was used in 80.1% of consultations, and the CRP test in 80.9%.

Patients aged 65+ had a higher probability of being diagnosed with pneumonia than those younger than 18 years (M2: Odds ratio (OR) 3.86, 95% confidence interval (CI) 1.48–10.08) (Table 2). Sore throat was negatively associated with being diagnosed with pneumonia (M2: OR 0.29, CI 0.15–0.55). Fever was associated with being diagnosed with pneumonia in M1 (OR 2.25, CI 1.28–3.94), while no association with fever as a single parameter was found in M2 (OR 0.39, CI 0.11–1.39). In contrast, the combination of fever and cough was significantly associated (M2: OR 11.10, CI 2.87–42.97). The clinical findings of poor general condition (M2: OR 5.96, CI 2.43–14.60), tachypnoea (M2: OR 2.55, CI 1.38–4.73) and abnormal lung auscultation (M2: OR 11.41, CI 4.14–31.45) were all associated with being diagnosed with pneumonia.

The median odds ratio (MOR) of the second-level (physicians) was 4.50 (M2).

## Discussion

### Key results

When differentiating pneumonia from acute bronchitis/bronchiolitis, common cold/influenza, and COVID-19, out-of-hours physicians mainly considered the patients’ clinical condition, respiratory rate and lung auscultation. Also, age over 65 seemed to be important in this assessment.

### Comparison with existing literature

Primary care physicians have been found to have good diagnostic accuracy for pneumonia when they are sure of the diagnosis after clinical assessment [20]. Moreover, the overall clinical impression is often used as a primary tool for diagnostic purposes [11]. The COVID-19 pandemic brought new diagnostic challenges, with an infection holding various symptoms and clinical findings that may resemble pneumonia [18]. Hence, the overall clinical impression was probably crucial for diagnosing pneumonia even during the late phase of the pandemic.

Despite questionable quality, the SARS-CoV-2 POCTs were widely and equally used in consultations for pneumonia and COVID-19 in our material [8]. Some COVID-19 patients may have been tested with PCR or SARS-CoV-2 POCT before the consultation, which was not included in the APO chart. The high usage of POCTs illustrates the challenge of distinguishing the two diagnoses and how high availability tends to increase the use of POCTs [21, 22]. A positive test result from a pre-taken PCR or SARS-CoV-2 POCT may have convinced the physicians of a COVID-19 diagnosis regardless of symptoms or clinical findings.

In a pre-pandemic study using the APO method in general practice, patients diagnosed with pneumonia more frequently had fever, dyspnoea, and increased purulent sputum than those diagnosed with bronchitis [23]. These findings differ slightly from what we found in the regression with combinations of symptoms (M2). Here, cough was negatively associated with pneumonia, and fever showed no association (M2), while the combination of fever and cough was highly significant. These results indicate that the physicians considered that fever or cough alone had a low predictive value for pneumonia, while fever and cough in combination had a high predictive value. The former study also reported a correlation with healthcare personnel assessing the patient as unwell. This corresponds well with our finding of an association with the physician assessing the patient’s general condition as poor. The differences between the findings of the two studies may be due to different APO charts, different clinical settings, or pre-pandemic versus pandemic periods. We hold it likely that all three factors contributed.

Adventitious respiratory sounds are present in healthy people [24]. Moreover, lung auscultation has low sensitivity for pneumonia, and one could argue that better diagnostic modalities should replace auscultation when possible [25]. Nevertheless, primary care physicians still seem to rely on abnormal pulmonary auscultation in differentiating between bronchitis and pneumonia [23]. We found that abnormal lung auscultation was associated with the pneumonia diagnosis, but in combination with fever, this correlation was extinguished. From a clinical point of view, this finding is surprising. A confounding factor may explain it, as the APO charts had a restricted number of clinical variables. The unknown results from POCTs may be one such factor.

Spanish and Danish GPs differed in distinguishing pneumonia from other lower RTIs, with a higher prevalence of pneumonia in Denmark [26]. Even in our material, there seems to be a marked difference in the percentage of pneumonia diagnoses between the five countries, ranging from 6.6% in Spain to 33.3% in Lithuania. These differences may be due to organisational factors influencing which healthcare services patients seek for different clinical problems. They may also come from cultural variations in physicians’ assessments. The finding of a median odds ratio of 4.50 (M2) of the physician level in the logistic regression supports this viewpoint. A median odds ratio of 4.50 means that when comparing two identical patients from randomly selected physicians, the odds of being diagnosed with pneumonia for a patient from the physician with the highest probability to diagnose pneumonia (say physician A) is 4.50 times the odds of a pneumonia diagnosis from the physician with the lowest probability (say physician B) [27]. If we randomly select pairs of doctors many times, the odds of pneumonia would be less than 4.50 for a patient of physician A versus physician B in half of the comparisons and higher than 4.50 in half.

We found a low prevalence of pneumonia among the youngest children compared to findings in general practice and hospitals [23, 28]. Parents had access to paediatric wards with direct attendance as an alternative for their children in some of the geographical areas in our study. This organisational factor may explain the low prevalence of pneumonia among the youngest children and why several physicians mainly registered adult patients.

During the COVID-19 pandemic, remote consultations have increased dramatically [29]. Our results indicate that pneumonia is diagnosed based on clinical signs more than symptoms. This result gives weight to arguments for physical consultations for respiratory tract infections, and one may question if prescribing antibiotics in remote RTI consultations should occur.

### Strengths and limitations

This study has some limitations. The data were collected through physicians’ self-registrations of a pre-defined set of clinical information on the APO charts. Self-registering risks compromising internal validity. However, the participants were to work on their registrations later during follow-up meetings, so they had no incentive for poor or false recording. Selection bias from recruiting physicians interested in respiratory tract infections and quality improvement may compromise external validity. The APO method has been used and deemed reliable in several studies in multiple countries [30–32]. A strength of this method is that registering one’s practice takes place in a regular working environment. Hence, despite the mentioned limitations, we believe the APO charts mirror the clinical reality we aim to investigate.

The APO chart was not made for the specific purpose of this study but to make the out-of-hours physicians register symptoms that could explain their antibiotic prescribing. As pneumonia is a diagnosis that most often requires antibiotic treatment, we believe the registrations are suitable even for analysing the diagnostic process. A limitation of the cross-sectional design of this study is that it does not permit an investigation of causality. There is a risk that the out-of-hours physicians decide to treat a patient with an RTI with antibiotics and, therefore, diagnose pneumonia instead of vice versa. Such subjectivity cannot be discerned in this study.

Merging influenza and the common cold into one category on the APO chart is a limitation. The two diagnoses have an overlapping clinical picture, but one would expect the combination of cough and fever to be more frequent and pronounced in influenza than in the common cold. The correlation between this symptom combination and pneumonia could become negligible if the regression included influenza as a separate category. However, we consider that most patients in the merged category had the common cold and that this weakness is less critical in interpreting the results.

The choice of not including any POCT results on the APO charts was made for two reasons. The first was the a priori assumption of low and variable use across the five countries. The second was for feasibility, considering the risk of lacking and inaccurate registration of the results. The registration of POCT use solely is a significant limitation, as the results could have been essential variables in the analyses aiming to explain the diagnostic processes [33]. Such results would be particularly interesting for the SARS-CoV-2 POCT.

European out-of-hours care has diverse organisational models between countries and within countries [17]. The organisational differences between the five countries in this study represent a methodological challenge, but it also increases the generalisability across the different European out-of-hours organisations.

## Conclusions

Diagnosing pneumonia, among other RTIs in out-of-hours services during the late phase of the COVID-19 pandemic, was associated with the symptom combination of cough and fever and the physicians’ evaluation of patients’ general condition, respiratory rate, and lung auscultation findings. Notably, the pneumonia diagnosis seems to have been mainly based on clinical assessments, and the risk of over- and underdiagnosing is imminent. Hence, future research on the diagnostic process of RTIs in out-of-hours primary care should include the results of POCTs and the aid of more accurate diagnostic modalities.

## Supplementary Information

Supplementary Material 1.

## Data Availability

All open results from HAPPY PATIENT are stored in an Open Access repository, the Zenodo platform (http://zenodo.org). Zenodo is an EU-backed portal linked to the OpenAIRE initiative (https://www.openaire.eu/, with a Digital Object Identifier (DOI) system (http://www.doi.org). All open data are also available on the project webpage (https://happypatient.eu/).

## Abbreviations

RTI: Acute respiratory tract infection
OR: Odds ratio
CI: 95% confidence interval
POCT: Point-of-care tests
CRP: C-reactive protein
APO: Audit Project Odense
IQR: Interquartile range
M1: Model 1
M2: Model 2
