# Supplementary material for: Diagnosing acute lower respiratory tract infections in out-of-hours services during the COVID-19 pandemic
Source: Int J Emerg Med. 2025 Jul 30;18:138. doi: 10.1186/s12245-025-00942-z (PMC12312447; doi:10.1186/s12245-025-00942-z)
Supplement: Supplementary file 1 — Supplementary Material 1. [file 12245_2025_942_MOESM1_ESM.pdf]

HAPPY PATIENT

ID: *9-9-999*

HAPPY PATIENT-1:
Suspected acute respiratory- or urinary tract infections in OOH-services

HAPPY PATIENT

	Age (years)	Sex		Du-ration	Symptoms									Clinical findings					Tests performed					Diagnosis											Antibiotics? (only systemic)											AB duration	Admit-tance	
					min 1 X									min 1 X					min 1 X					min 1 X											min 1 X												only 1 X	
				Number of days with symptoms (99=unknown)	Fever (temp. ≥ 38 °C)	Sore throat / throat pain	Cough	Purulent sputum / increased sputum production	Ear pain	Dysuria, frequency or urgency	Flank/back pain	Other symptoms	Poor general condition (incl. confusion)	Tonsillar exudates	Tender cervical adenopathy	Tachypnoea	Abnormal lung auscultation	None of the above	Strep-A test performed	Urinary dipstick performed	CRP test performed	COVID-19 test performed	None of the above	COVID-19	Common cold / influenza	Acute otitis media	Acute rhinosinusitis	Acute pharyngo-tonsillitis	Acute bronchitis/bronchiolitis	Pneumonia	COPD exacerbation	Cystitis	Pyelonephritis	None of the above	No antibiotics	Penicillin V or pivmecillinam	Amoxicillin	Amoxicillin + clavulanic acid	Fosfomycin	Nitrofurantoin	Trimethoprim +/- sulfonamide	Macrolides or clindamycin	Cephalosporins	Quinolones	Other antibiotics	Antibiotic treatment duration – days (999=unknown 0=no antibiotics)	Admitted to hospital	Not admitted to hospital
		F	M	1	2	3	4	5	6	7	8	9	10	11	12	13	14	15	16	17	18	19	20	21	22	23	24	25	26	27	28	29	30	31	32	33	34	35	36	37	38	39	40	41	42	43	44	45
1																																																
2																																																
3																																																
4																																																
5																																																
6																																																
7																																																
8																																																

Final version

OOH-UK

®Copyright: Audit Projekt Odense, J.B. Winsløws Vej 9A, 1. 5000 Odense C
